# Factorial validation and psychometric evaluation of the Arabic early childhood oral health impact scale among Saudi mothers

**DOI:** 10.3389/froh.2026.1923290

**Published:** 2026-10-20

**Authors:** Nourh I. Alissa, Hissah Z. AlZamil

**Affiliations:** Department of Orthodontics and Pediatric Dentistry, College of Dentistry, Qassim University, Buraydah, Saudi Arabia

**Keywords:** Arabic, early childhood caries, ECOHIS, factorial validation, oral health-related quality of life, preschool children, child

## Abstract

**Background:**

The Arabic version of the Early Childhood Oral Health Impact Scale (A-ECOHIS) has been shown to have acceptable reliability and basic validity in previous work, but no study has yet examined its factor structure using advanced psychometric methods with independent samples for exploratory and confirmatory analyses. The objective of the present study is to evaluate the factorial validity of the A-ECOHIS by conducting both exploratory and confirmatory factor analyses in a large group of Saudi mothers with preschool-aged children.

**Methods:**

A cross-sectional study was conducted among 602 mother-child (children aged 2–6 years) recruited through a combination of convenience and snowball sampling. Mothers completed the 13-item A-ECOHIS via an online questionnaire that was distributed via email to educational institutions and shared on a social media platform (WhatsApp). The total sample was randomly split into two independent subsamples; after listwise deletion; the analytic samples were (*n* = 285) for exploratory factor analysis (EFA) and (*n* = 286) for confirmatory factor analysis (CFA). To assess internal consistency, Cronbach's alpha and McDonald's omega were calculated. The suitability of the data for factor analysis was examined using the Kaiser-Meyer-Olkin test and Bartlett's test of sphericity. EFA was then run, employing principal axis factoring with Oblimin rotation based on a polychoric correlation matrix. CFA was performed using WLSMV (Weighted Least Squares Mean and Variance Adjusted) estimation in R with the lavaan package on the independent holdout sample to account for the ordinal nature of the ECOHIS items. Convergent validity was evaluated using the average variance extracted (AVE) and composite reliability (CR), along with Spearman's correlation between the A-ECOHIS and Child Oral Health Rating. Discriminant validity was assessed using the Fornell-Larcker criterion and the HTMT ratio.

**Results:**

The overall scale demonstrated good internal consistency (Cronbach's *α* = 0.825; McDonald's *ω* = 0.827) and the provisional 11-item scale showed similarly good reliability (*α* = 0.828, *ω* = 0.828). EFA yielded a four-factor solution (Family Impact, Child Self-Image, Child Psychology, and Child Function and Symptoms), which together explained 57.76% of the total variance. CFA on the independent Sample 2 using WLSMV estimation supported this four-factor structure, showing acceptable model fit: CFI = 0.979, TLI = 0.969, RMSEA = 0.070 (90% CI: 0.052–0.088), and SRMR = 0.049. AVE values were 0.625 for Family Impact, 0.850 for Child Self-Image, 0.551 for Child Psychology, and 0.642 for Child Function and Symptoms. Composite reliability (CR) values were 0.869, 0.919, 0.709, and 0.843, respectively. A significant correlation between total ECOHIS score and Child Oral Health Rating (Spearman's rho = 0.443, *p* < 0.001) supported external convergent validity. Two items (pronunciation difficulty and missed school) did not load on any factor.

**Conclusion:**

The Arabic A-ECOHIS demonstrated a four-factor structure in this Saudi maternal sample, differing from the original six-domain model. The provisional 11-item model showed acceptable fit in the independent holdout sample. However, the Child Psychology subscale showed questionable reliability, and two items were not retained. Therefore, it should be considered preliminary rather than a validated alternative. Further psychometric refinement and external validation in newly recruited independent samples are warranted.

## 1 Introduction

Early childhood caries (ECC) is a fast-moving, multifaceted condition shaped by a mix of biological, behavioral, and social factors (1, 2). Defined as the presence of one or more decayed, missing, or filled tooth surfaces in primary teeth among children aged 71 months or younger, ECC represents a major global public health challenge (3). A systematic review and meta-analysis reported a global prevalence of ECC of approximately 50% (4). In Saudi Arabia, the burden is particularly alarming; recent meta-analyses have documented prevalence rates of 75.4% in primary dentition, with individual studies reporting rates ranging from 27% to 77% among preschool children across various regions of the Kingdom (5–8). A 2024 hospital-based study in Riyadh found that 76% of children aged 36–71 months had ECC (9).

Beyond the clinical sequelae of pain and functional impairment, ECC exerts profound detrimental effects on the oral health-related quality of life (OHRQoL) of affected children and their families. OHRQoL encompasses physical, psychological, and social well-being and has emerged as a critical outcome for evaluating the true burden of oral disease. Untreated caries has been shown to have a significant positive association with impaired OHRQoL (10). The impact of ECC extends beyond the child and family, increasing the overall burden on communities. Importantly, how parents perceive and understand their child's oral health has a substantial impact on the likelihood that the child will develop early childhood caries (2, 11).

To systematically evaluate these effects, Pahel et al. constructed the Early Childhood Oral Health Impact Scale (ECOHIS), a 13-item questionnaire designed to measure the caregiver's perception of OHRQoL in preschool children. The instrument is divided into two main parts: the Child Impact Section (9 items), covering symptoms, functioning, psychological well-being, and self-image/social interaction, and the Family Impact Section (4 items), covering parental distress and family functioning. The original ECOHIS showed good psychometric properties, with Cronbach's alpha of 0.91 for the child section and 0.95 for the family section, and an intraclass correlation coefficient (ICC) of 0.84 for test-retest reliability (12). The scale has since been translated and validated across numerous languages and cultural contexts, including French, Chinese, Brazilian Portuguese, Spanish, Lithuanian, Malay, and Farsi (13–19).

Farsi et al. previously translated the ECOHIS into Arabic and validated the resulting A-ECOHIS. Their work demonstrated good reliability, with a Cronbach's alpha of 0.85 for the total scale and an intraclass correlation coefficient of 0.86, along with supportive evidence for both convergent and discriminant validity. However, a critical methodological limitation must be acknowledged: Farsi et al.'s validation did not employ advanced psychometric techniques to examine the scale's underlying factor structure. More specifically, while their validation work examined internal consistency, test-retest reliability, and construct validity by comparing scores across known groups, it did not include either exploratory or confirmatory factor analysis (20). Consequently, whether the original two-factor (child and family) or six-domain structure of the ECOHIS is applicable to Arabic-speaking populations remains empirically unsubstantiated.

Establishing factorial validity is not a trivial exercise; it is essential for confirming that scale items indeed load onto the theoretically intended domains, thereby justifying the calculation and interpretation of domain-specific and total scores. Without this evidence, the validity of conclusions drawn from A-ECOHIS scores in research and clinical practice remains incomplete. Other language versions have been subjected to factor analysis; for example, the Farsi version was found to have a three-dimensional structure rather than the hypothetical six-domain model (19), highlighting the need for similar rigorous testing of the Arabic version. As the A-ECOHIS gains increasing use in national studies, rigorous factorial validation has become an urgent methodological necessity.

The present study, therefore, aimed to conduct a thorough psychometric evaluation of the Arabic version of the Early Childhood Oral Health Impact Scale (A-ECOHIS) using a factor-analytic approach incorporating exploratory and confirmatory factor analyses in a large sample of Saudi mothers with their preschool-aged children. This study provides researchers and clinicians with the necessary evidence to confidently interpret A-ECOHIS scores, thereby enhancing the accuracy of OHRQoL assessments in early childhood oral health research across Saudi Arabia and other Arabic-speaking countries.

## 2 Methods

### 2.1 Study design and participants

This study used a cross-sectional design, carried out in Saudi Arabia. The participants were mothers whose children were between the ages of two and six years old across several regions in the country including Riyadh region, Makkah region, Madinah region, Aseer region, Eastern region, Al-Qassim, and Tabuk region. In the current study, the age range was selected based on the age range used in the original Farsi et al. validation study of the A-ECOHIS, in which the questionnaire was validated among children aged 6 years or younger (≤72 months) (20). Mothers were included if they gave their consent and completed the online questionnaire. We excluded those who were not Saudi nationals, who had more than two items missing in the child section or more than one in the family section, whose children had systemic illnesses or genetic syndromes, and any questionnaires that were filled out by the father rather than the mother.

Participants were recruited through a combination of convenience and snowball sampling. The questionnaire link was distributed via email to educational institutions and shared on a social media platform (WhatsApp) in groups dedicated to schools, parents, and parent associations. The online survey was created using Google Forms and was available in Arabic.

### 2.2 Sample size estimation

The required sample size was calculated using a 95% confidence level and a 5% margin of error. Based on an estimated total population of 130,000 preschool children in Saudi Arabia, a minimum sample of 385 participants was deemed necessary. To account for potential incomplete responses and dropouts from the online distribution method, the target sample size was increased to 450 participants. A total of 602 eligible mothers completed the questionnaire, meeting the required sample size for both exploratory and confirmatory factor analyses.

In addition to the prevalence-based calculation, the sample size was justified based on established guidelines for factor analysis. For EFA, a minimum of 5–10 participants per item is recommended, requiring at least 130 participants for the 13-item scale. For CFA, a sample size of ≥ 200 is recommended for stable parameter estimation and adequate statistical power. To enable independent subsamples for EFA and CFA, a target sample size of at least 500 participants was set, following recommendations for split-sample cross-validation in psychometric studies.

The total sample (*N* = 602) was randomly split into two equal halves to ensure independent samples for EFA and CFA. Sample 1 (*n* = 301) was used for the exploratory factor analysis (EFA), and Sample 2 (*n* = 301) was used for the confirmatory factor analysis (CFA). This split-sample approach ensures that the factor structure identified in the EFA is tested on an independent sample, providing a more rigorous test of factorial validity.

### 2.3 Questionnaire and data collection

The final questionnaire consisted of three sections. The first section included a brief introduction clarifying the study objectives and procedures, followed by an informed consent statement. Participants could not proceed to the subsequent sections without providing consent. The second section included verification of inclusion criteria and sociodemographic questions, including the child's age and gender, the mother's and father's ages, and parental education levels. The questionnaire also featured a single global item asking parents, “In general, how would you rate the overall oral health of your child?” with response options: Excellent, Average, Poor, and I don't know. Responses of “I don't know” were treated as missing, consistent with the handling of missing data in the ECOHIS. This explains why the Child Oral Health Rating variable has *n* = 586 instead of 602 (16 participants selected “I don't know”). The third section consisted of the Arabic version of the Early Childhood Oral Health Impact Scale (A-ECOHIS).

The A-ECOHIS had been previously translated into Arabic and validated for reliability and basic validity (20). The scale contains 13 items organized into two major sections. The Child Impact Section has nine items covering child symptoms (one item), function (four items), psychology (two items), and self-image/social interaction (two items). The Family Impact Section includes four items addressing parental distress (two items) and family function (two items). Each item uses a five-point Likert scale: 0 = never, 1 = rarely ever, 2 = sometimes, 3 = often, 4 = very often. A “don't know” option is also available and is treated as a missing value. Participants were excluded if they had more than two items missing in the child section or more than one in the family section. For score-based analysis, ECOHIS total, section and domain scores were calculated only for participants with complete responses to all the 13 ECOHIS items no imputation was performed. The total A-ECOHIS score ranges from 0 to 52 (child section: 0–36; family section: 0–16), with lower scores reflecting better oral health-related quality of life. The link to the online questionnaire was made available from April through October of 2019.

### 2.4 Statistical analysis

Data were processed in Excel and analyzed using IBM SPSS (version 26), IBM SPSS Amos (version 26), R with the lavaan and semTools packages (version 0.7-2 and version 0.5–8), and JASP (version 0.19). Categorical variables were described as frequencies and valid percentages, while continuous variables were summarized using mean, standard deviation (SD), minimum, maximum, skewness, and kurtosis.

Missing data were handled as follows: “I don't know” responses were treated as missing, consistent with the original ECOHIS validation (12). Item-level descriptive analysis and floor effect were calculated using available responses. For the analysis of ECOHIS total, section or domain scores, analysis was applied only for complete cases; only participants with responses to all 13 ECOHIS items were included (*n* = 567). For factor analyses (EFA and CFA), listwise deletion was applied; sample 1 was *n* = 285 and sample 2 *n* = 286. The proportion of missing data was minimal (< 5% across all items). Missing responses per ECOHIS item (including “I don't know” responses treated as missing) and floor effects were calculated.

Floor effects were calculated as the percentage of respondents scoring the minimum value (0 = “Never”) for each ECOHIS item. Floor effects were categorized as high (> 50%), moderate (30%–50%), or low (< 30%).

#### 2.4.1 Reliability analysis

To evaluate the internal consistency of the Arabic ECOHIS, we calculated Cronbach's alpha coefficients. Reliability was assessed for the full 13-item scale, the nine-item Child Impact Section, the four-item Family Impact Section, and each individual domain, namely, Child Function, Child Psychology, Child Self-Image, Parental Distress, and Family Function. We also computed corrected item-total correlations. Following EFA, Cronbach's alpha and McDonald's omega were recalculated for the empirically derived factor structure and its retained items. Cronbach's alpha values were interpreted using standard thresholds: 0.90 or above was considered excellent, 0.80 to 0.89 good, 0.70 to 0.79 acceptable, 0.60 to 0.69 questionable, and 0.59 or below poor (21, 22).

To assess reliability more comprehensively for ordinal data, we calculated McDonald's omega (*ω*) for each factor and the total scale. Additionally, Composite Reliability (CR) was calculated for each factor derived from the confirmatory factor analysis to assess construct reliability.

#### 2.4.2 Factor analysis

The Kaiser-Meyer-Olkin measure and Bartlett's test of sphericity were used to asses the suitability for factor analysis (23). For the EFA, these statistics were calculated from the polychoric correlation matrix used for factor extraction.

##### 2.4.2.1 Sample split for EFA and CFA

To ensure independent samples for exploratory and confirmatory analyses, the total sample (*N* = 602) was randomly split into two equal halves using a random number generator with a fixed seed for reproducibility. Sample 1 (*n* = 301) was used for the EFA, and Sample 2 (*n* = 301) was used for the confirmatory factor analysis (CFA).

##### 2.4.2.2 Exploratory factor analysis (EFA)

For the EFA, we applied Principal Axis Factoring (PAF) along with Oblimin rotation on Sample 1 (*n* = 285), since we expected the extracted factors to be correlated. A polychoric correlation matrix was estimated from the ordinal ECOHIS items in Sample 1 (*n* = 285). The number of factors to retain was guided by parallel analysis and visual inspection of the scree plot (24). Loadings ≥ 0.40 were considered salient for factor interpretation (25).

###### 2.4.2.2.1 Parallel analysis

To determine the number of factors to retain, Horn's parallel analysis was performed on the EFA sample (Sample 1, *n* = 285), using the polychoric correlation matrix and FA-based extraction with 1,000 simulated datasets. Factors were retained if the real data eigenvalue exceeded the simulated data mean eigenvalue (26, 27).

##### 2.4.2.3 Confirmatory factor analysis (CFA)

We performed CFA using WLSMV (Weighted Least Squares Mean and Variance Adjusted) estimation in R with the lavaan package (version 0.7-2) on Sample 2 (*n* = 286) to account for the ordinal nature of the ECOHIS items. The ordered=TRUE argument was used to treat the Likert-scale items as ordinal, which automatically invokes WLSMV estimation with polychoric correlations. Good model fit was defined as CFI ≥ 0.90, TLI ≥ 0.90, RMSEA ≤ 0.08, and SRMR ≤ 0.08.

Modification indices were examined to identify potential sources of model misspecification. A correlated residual was tested between ‘Family member took time off work‘ and ‘Financial impact on the family’ (MI = 13.26), because both items assess practical family consequences of the child's dental problems.

###### 2.4.2.3.1 Competing models

To evaluate the adequacy of the proposed four-factor structure, we compared it against two competing models using CFA on Sample 2 (*n* = 286): (1) a one-factor model with all 11 items loading on a single OHRQoL factor, and (2) a two-factor model consistent with the original ECOHIS structure (Child Impact and Family Impact). Model comparison was based on comparative fit indices, including CFI/TLI and RMSEA values.

###### 2.4.2.3.2 Convergent and discriminant validity

To check convergent validity, we used the Average Variance Extracted (AVE), considering values above 0.50 as evidence of adequate convergence (28). Composite Reliability (CR) was calculated for each factor, with values above 0.70 indicating adequate construct reliability.

Discriminant validity was assessed using two complementary approaches: the Fornell-Larcker criterion (where the square root of the AVE for each factor should exceed the inter-factor correlations) and the HTMT (Heterotrait-Monotrait) ratio, was calculated using the senTools package. with values below 0.85 considered adequate discriminant validity.

#### 2.4.3 Convergent validity (external)

To assess external convergent validity, we calculated Spearman's rank-order correlation between the total ECOHIS score and the Child Oral Health Rating (a single global item: “In general, how would you rate the overall oral health of your child?”). The original response categories were Excellent, Average, Poor, and I don't know. Correlation analysis included only participants with complete responses to all 13 ECOHIS items and a valid Child Oral Health Rating, resulting in *n* = 553.

### 2.5 Ethical consideration

The study protocol received ethical clearance from the Institutional Review Board at Qassim University (approval reference [Code#: ST/58/2019], issued on [17/3/2019]). Prior to completing the questionnaire, each participant provided electronic informed consent.

## 3 Results

### 3.1 Descriptive statistics

A total of 730 questionnaires were received in this study; 128 were excluded (questionnaire filled by fathers, age > 72 months and missing values in child and family domain). The mean child age was 4.94 years (SD = 1.02), ranging from 2 to 6 years, corresponding to 59.3 months (SD = 12.3, range from 24 to 72 months). The sample was balanced by gender (50.2% female, 49.8% male). Most children attended preschool (83.9%), 16.1% of the children did not attend kindergarten were eligible for inclusion to broaden the range of recruitment sources and provided that they met the same predefined eligibility criteria as the other participants. Most mothers held a bachelor's degree (64.8%) and were aged 26–35 years (59.0%). Regarding the Child Oral Health Rating, 30.2% rated their child's oral health as excellent, 55.8% as average, and 14.0% as poor. Sixteen participants (2.7%) selected “I don't know” and were treated as missing, resulting in a valid *n* of 586 for this variable. All details are shown in Table 1.

**Table 1:** Demographic characteristics (*n* = 602).

Characteristic	Value
Child Characteristics	
Child age in years, mean (SD)	4.94 (1.02)
Child age in years, range	2–6 years
Child age in months, mean (SD)	59.3 (12.3)
Child age in months, range	24–72 months

Child gender	*n (%)*
Female	302 (50.2%)
Male	300 (49.8%)

Child attending preschool	*n (%)*
Yes	505 (83.9%)
No	97 (16.1%)

Child Oral Health Rating (*n* = 586)	*n (%)*
Excellent	177 (30.2%)
Average	327 (55.8%)
Poor	82 (14.0%)
Mother Characteristics	

Mother age (years)	*n (%)*
≤25	13 (2.2%)
26–35	355 (59.0%)
36–45	214 (35.5%)
≥46	20 (3.3%)

Education level	*n (%)*
High school	125 (20.8%)
Bachelor's degree	390 (64.8%)
Post graduate	38 (6.3%)
Other	49 (8.1%)

Percentages are valid percent (excluding missing values).

Among individual items, “missed daycare or preschool” was the most frequently reported impact (43.5% reported sometimes or more, where 0.2% reported very often, 5.9% reported often, and 37.4% reported sometimes), followed by “irritable or frustrated” (42.7%, where 0.8% reported very often, 7.3% reported often, and 34.6% reported sometimes) and “pain” (37.5%, where 0.8% reported very often, 6.6% reported often, and 30.1% reported sometimes). The least frequently reported impacts were “avoided smiling or laughing” (13.9%) and “avoided talking” (15.6%), as shown in Table 2.

**Table 2:** Frequency distribution of ECOHIS items (*n* = 602).

Item, *n* (%)	Very often	Often	Sometimes	Rarely	Never
Child Impact Section					
Pain in the teeth, mouth, or jaws	5 (0.8)	40 (6.6)	181 (30.1)	197 (32.7)	179 (29.7)
Difficulty drinking beverages	14 (2.4)	0 (0)	127 (21.3)	185 (31.1)	269 (45.2)
Difficulty eating certain foods	5 (0.8)	30 (5.0)	161 (27.0)	182 (30.5)	219 (36.7)
Difficulty pronouncing words	10 (1.7)	33 (5.5)	135 (22.5)	172 (28.7)	250 (41.7)
Missed daycare/preschool	1 (0.2)	35 (5.9)	223 (37.4)	198 (33.2)	139 (23.3)
Trouble sleeping	2 (0.3)	20 (3.3)	126 (21.0)	215 (35.8)	238 (39.6)
Irritable or frustrated	5 (0.8)	44 (7.3)	207 (34.6)	166 (27.7)	177 (29.5)
Avoided smiling/laughing	1 (0.2)	4 (0.7)	77 (13.0)	135 (22.7)	377 (63.5)
Avoided talking	0 (0)	10 (1.7)	83 (13.9)	140 (23.5)	364 (61.0)
Family Impact Section					
Family member upset	9 (1.5)	50 (8.4)	125 (20.9)	149 (24.9)	265 (44.3)
Family member felt guilty	18 (3.0)	77 (12.8)	144 (24.0)	133 (22.2)	228 (38.0)
Taken time off work	0 (0)	20 (3.3)	88 (14.7)	144 (24.0)	348 (58.0)
Financial impact on the family	14 (2.3)	50 (8.3)	119 (19.8)	122 (20.3)	297 (49.3)

Percentages are valid percent (excluding missing values).

Thirty-five participants (5.8%) had at least one missing ECOHIS item and were therefore excluded from the score-based analyses. The remaining 567 participants had complete ECOHIS responses and were included in these analyses. Missing responses per item ranged from 0% to 1.3% across the 13 ECOHIS items (Table 3). The highest missing rates were observed for “avoided smiling or laughing” (1.3%), “difficulty drinking hot/cold beverages” (1.2%), and “missed daycare or preschool” (1.0%). All other items had missing rates below 1%. Item-level missingness was low (0%–1.3%).

**Table 3:** Missing responses and floor effects for ECOHIS items (*n* = 602).

Item	Valid n	Missing *n* (%)	Never (n)	Floor Effect (%)	Interpretation
Pain in the teeth, mouth, or jaws	602	0 (0.0%)	179	29.7%	Low
Difficulty drinking beverages	595	7 (1.2%)	269	45.2%	Moderate
Difficulty eating certain foods	597	5 (0.8%)	219	36.7%	Moderate
Difficulty pronouncing words	600	2 (0.3%)	250	41.7%	Moderate
Missed daycare/preschool	596	6 (1.0%)	139	23.3%	Low
Trouble sleeping	601	1 (0.2%)	238	39.6%	Moderate
Irritable or frustrated	599	3 (0.5%)	177	29.5%	Low
Avoided smiling/laughing	594	8 (1.3%)	377	**63**.**5%**	**High** *
Avoided talking	597	5 (0.8%)	364	**61**.**0%**	**High** *
Family member upset	598	4 (0.7%)	265	44.3%	Moderate
Family member felt guilty	600	2 (0.3%)	228	38.0%	Moderate
Taken time off work	600	2 (0.3%)	348	**58**.**0%**	**High** *
Financial impact on the family	602	0 (0.0%)	297	49.3%	Moderate

Missing responses include “I don't know” responses and blank responses.

* Floor effect = percentage scoring the minimum value (0 = “Never”). Floor effects > 50% are considered high, 30%–50% moderate, and < 30% low.

Among the 567 participants who completed all the ECOHIS responses, the mean total ECOHIS score averaged 12.25 (SD = 7.17), with individual scores ranging from 0 to 33. The Child Impact Section had a mean of 8.53 (SD = 4.91), whereas the Family Impact Section showed a mean of 3.72 (SD = 3.27). Skewness and kurtosis values for all scores remained within ±2, indicating the data were acceptably normal for parametric testing (see Table 4**)**.

**Table 4:** ECOHIS domain and total scores among complete cases (*n* = 567).

Category	Mean	SD	Min	Max	Skewness	Kurtosis
Total ECOHIS Score (0–52)	12.25	7.17	0	33	0.364	−0.518
Child Impact Section (0–36)	8.53	4.91	0	21	0.363	−0.567
Child Symptom Domain (0–4)	1.16	0.95	0	4	0.306	−0.749
Child Function Domain (0–16)	4.22	2.72	0	12	0.525	−0.311
Child Psychology Domain (0–8)	2.09	1.56	0	8	0.335	−0.469
Child Self-Image Domain (0–8)	1.07	1.42	0	6	1.047	0.097
Family Impact Section (0–16)	3.72	3.27	0	13	0.612	−0.464
Parental Distress Domain (0–8)	2.16	1.97	0	8	0.571	−0.583
Family Function Domain (0–8)	1.56	1.73	0	7	0.853	−0.303

Scores were calculated only for participants with complete responses to all 13 ECOHIS items.

### 3.2 Reliability assessment

#### 3.2.1 Cronbach's alpha testing

The original Arabic ECOHIS demonstrated good internal consistency, with a Cronbach's *α* of 0.825 for the total scale. The original Child Impact Section demonstrated acceptable reliability, with a Cronbach's alpha of 0.756, and the Family Impact Section also showed acceptable consistency (*α* = 0.794). The Child Self-Image, had the highest internal consistency (*α* = 0.831, *ω* = 0.832), while Child Function domain (drinking, eating, pronunciation, and missed daycare/preschool) displayed a poor reliability alpha (*α*= 0.590, *ω* = 0.645). For the final four-factor, 11-item structure, the total scale demonstrated good internal consistency (*α* = 0.828, *ω* = 0.828). Family Impact, four items, showed acceptable reliability (*α* = 0.794, *ω* = 0.796), similarly to the original ECOHIS. Child Self-Image, two items, had the highest internal consistency (*α* = 0.831, *ω* = 0.832). In contrast to the original Child function domain, the Child Function and Symptoms, with the three items: pain, drinking and eating, showed acceptable reliability (*α* = 0.762, *ω* = 0.771). While Child Psychology, two items, had a Cronbach's alpha of 0.614, which is considered questionable according to our predefined criteria Table 5. Corrected item-total correlations ranged from 0.296 to 0.570. The items “difficulty pronouncing words” (r = 0.306) and “missed daycare or preschool” (r = 0.296) demonstrated lower correlations; however, their deletion did not materially improve the overall Cronbach's alpha as shown in Table 6**.** Their factorial performance was evaluated separately in the EFA Table 8**.**

**Table 5:** Cronbach's alpha for ECOHIS scales.

Scale	Number of Items	Valid n	Cronbach's *α*	McDonald's *ω*	Interpretation
Original 13-item A-ECOHIS	13	567	0.825	0.827	Good
Original Child Impact Section	9	574	0.756	0.758	Acceptable
Child Symptom^a^	Pain	602	NA	NA	-
Child Function	Drinking, eating, pronunciation, missed daycare	585	0.590	0.645	Poor ^b^
Child Psychology	Sleeping, irritable/frustrated	598	0.614	0.611	Questionable ^c^
Child Self-Image	Avoided smiling/laughing, avoided talking	592	0.831	0.832	Good
Original Family Impact Section	4	594	0.794	0.796	Acceptable
Parental Distress	Upset, guilty	596	0.737	0.736	Acceptable
Family Function	Time off work, financial impact	600	0.711	0.712	Acceptable
Provisional 11-item A-ECOHIS	11	573	0.828	0.828	Good
- Child Function & symptoms	Pain, drinking, eating	593	0.762	0.771	Acceptable
- Child Psychology	Sleeping, irritable/frustrated	598	0.614	0.611	Questionable ^c^
- Child Self-Image	Avoided smiling/laughing, avoided talking	592	0.831	0.832	Good
- Family Impact	Upset, guilty, Time off work, financial impact	594	0.794	0.796	Acceptable

a The Child Symptom domain consists of a single item (pain); Cronbach's α is not applicable.

b Cronbach's α ≤ 0.590 is interpreted as” poor” per study criteria.

c Per the study's criteria, values from 0.60–0.69 are considered “questionable”.

**Table 6:** Item-total cronbach's alpha statistics for the original 13-item A-ECOHIS (*n* = 567).

Domain	Corrected Item-Total Correlation	Cronbach's α if Item Deleted
Child Impact Section		
Pain in the teeth, mouth, or jaws	0.523	0.809
Difficulty drinking beverages	0.510	0.810
Difficulty eating certain foods	0.570	0.805
Difficulty pronouncing words	0.306	0.825
Missed daycare/preschool	0.296	0.825
Trouble sleeping	0.471	0.813
Irritable or frustrated	0.370	0.820
Avoided smiling/laughing	0.479	0.813
Avoided talking to other children	0.426	0.816
Family Impact Section		
Family member upset	0.567	0.805
Family member felt guilty	0.540	0.807
Family member taken time off work	0.544	0.808
Financial impact on the family	0.496	0.811

### 3.3 Factor analysis

#### 3.3.1 Checking the suitability of the questionnaire for factor analysis

For the EFA on Sample 1 (*n* = 285), the KMO was 0.797, and Bartlett's test was also significant (*χ*^2^ = 1,680.42, df = 78, *p* < 0.001). For the CFA on Sample 2 (*n* = 286), the KMO was 0.802, and Bartlett's test was also significant (*χ*^2^ = 1,257.44, df = 78, *p* < 0.001), supporting the suitability of the data for factor analysis.

#### 3.3.2 Exploratory factor analysis (EFA)

EFA based on the polychoric correlation matrix was performed using PAF with Oblimin rotation on Sample 1 (*n* = 285). The visual inspection of the scree plot suggested a four-factor solution. Followed by PAF extraction, the four retained factors accounted for 57.76% of the total variance **(**Table 7**)**. The exploratory factor analysis revealed a four-factor structure for the scale items. Factor 1 (Family Impact) primarily loaded the four family impact items: “financial impact on family” (0.787), “family member felt guilty” (0.723), “family member felt upset” (0.692), and “family member took time off work” (0.645). Factor 2 (Child Self-Image) strongly correlated with the two child self-image items: “avoided talking with other children” (1.050) and “avoided smiling or laughing” (0.692). Factor 3 (Child Psychology) was defined by two items: “trouble sleeping” (0.812) and “irritable or frustrated” (0.572). Factor 4 (Child Function and Symptoms) was associated with three items: “difficulty drinking beverages” (−0.778), “difficulty eating certain foods” (−0.613), and “pain in the teeth, mouth, or jaws” (−0.556). Pain also cross-loaded on Family Impact (0.434) but showed a larger absolute loading on Child Function and Symptoms. The two items (“difficulty pronouncing words” and “missed daycare or preschool”) did not load significantly on any factor (loadings < 0.40), as shown in Table 8. “The avoided-talking item” shown an ultra-Heywood estimate (loading = 1.050; communality = 1.058), indicating some local instability in the exploratory child Self-Image factor. This item nevertheless loaded on the same factor as avoided smiling/Laughing and was retained for subsequent confirmatory evaluation.

**Table 7:** Polychoric exploratory factor analysis summary on sample 1 (*n* = 285).

Statistics	Factor 1 (Family Impact)	Factor 2 (Child Self-Image)	Factor 3 (Child Psychology)	Factor 4 (Child Function and Symptoms)
SS Loadings	4.66	1.44	0.85	0.576
Proportion Var (%)	35.83	10.99	6.51	4.43
Cumulative Var (%)	35.83	46.82	53.33	57.76

**Table 8:** Factor loadings and variance explained for each factor of ECOHIS items in sample 1 (*n* = 285).

Item	Factor 1 (Family Impact)	Factor 2 (Child Self-Image)	Factor 3 (Child Psychology)	Factor 4 (Child Function and Symptoms)	Communality
Child Impact Section					
Pain in the teeth, mouth, or jaws	0.434^a^	−0.151	−0.020	−0.556 ^a^	0.645
Difficulty drinking hot/cold beverages	−0.030	0.125	0.089	−0.778	0.718
Difficulty eating certain foods	0.207	0.065	0.130	−0.613	0.667
Difficulty pronouncing words	0.022	0.173	0.320	−0.131	0.254
Missed daycare/preschool	−0.070	0.019	0.339	−0.181	0.181
Trouble sleeping	0.094	−0.078	0.812	−0.019	0.672
Irritable or frustrated	0.051	0.091	0.572	0.099	0.361
Avoided smiling/laughing	0.143	0.692	0.059	−0.072	0.641
Avoided talking to other children	−0.030	1.050^b^	−0.010	0.042	1.058
Family Impact Section					
Family member upset	0.692	0.147	−0.097	−0.103	0.585
Family member felt guilty	0.723	0.012	−0.043	−0.053	0.546
Family member taken time off work	0.645	0.040	0.064	−0.111	0.546
Financial impact on the family	0.787	−0.014	0.180	0.137	0.633

Extraction Method: Principal Axis Factoring based on a polychoric correlation matrix). Rotation Method: Oblimin.

a The pain item demonstrated cross-loading on Factor 1 (0.434) and Factor 4 (−0.556); it was assigned to Factor 4 based on theoretical interpretability and the higher absolute loading.

b The“avoided talking” item produced an ultra-Heywood estimate (loading = 1.050; communality = 1.058), indicating local instability in the exploratory solution.

Negative loadings reflect the orientation of the factor; absolute values are reported in the text.

The factor correlation matrix showed moderate correlations between factors (ranging from −0.443 to 0.461), supporting the use of Oblimin rotation and indicating that the factors are related but distinct constructs, as shown in Table 9.

**Table 9:** Factor correlation matrix from the polychoric EFA on sample 1 (*n* = 285).

Factor pair	Correlation
Factor 1 (Family Impact) vs. Factor 2 (Child Self-Image)	0.276
Factor 1 (Family Impact) vs. Factor 3 (Child Psychology)	0.303
Factor 1 (Family Impact) vs. Factor 4 (Child Function and Symptoms)	−0.443
Factor 2 (Child Self-Image) vs. Factor 3 (Child Psychology)	0.461
Factor 2 (Child Self-Image) vs. Factor 4 (Child Function and Symptoms)	−0.247
Factor 3 (Child Psychology) vs. Factor 4 (Child Function & Symptoms)	−0.399

##### 3.3.2.1 Parallel analysis

Parallel analysis was conducted on the EFA sample (Sample 1, *n* = 285) as shown in Table 10 and Figure 1, the first four eigenvalues (4.424, 1.051, 0.558, and 0.271) exceeded their corresponding simulated means (0.559, 0.297, 0.229, and 0.169, respectively). The fifth eigenvalue (0.071) fell below its simulated mean (0.116). Thus, parallel analysis supported a four-factor solution, consistent with the EFA findings and the CFA results, which confirmed the four-factor structure.

**Table 10:** Parallel analysis results for the arabic ECOHIS on sample 1 (*n* = 285).

Factor	Initial factor eigenvalues	Simulated Data Mean
Factor 1*	4.424	0.559
Factor 2*	1.051	0.297
Factor 3*	0.558	0.229
Factor 4*	0.271	0.169
Factor 5	0.071	0.116
Factor 6	−0.005	0.067
Factor 7	−0.044	0.019
Factor 8	−0.142	−0.028
Factor 9	−0.246	−0.074
Factor 10	−0.294	−0.120
Factor 11	−0.309	−0.169
Factor 12	−0.388	−0.221
Factor 13	−0.540	−0.286

* = Factor should be retained. Results from FA-based parallel analysis with 1,000 stimulated datasets on the EFA sample (Sample 1, *n* = 285).

**Figure 1:**
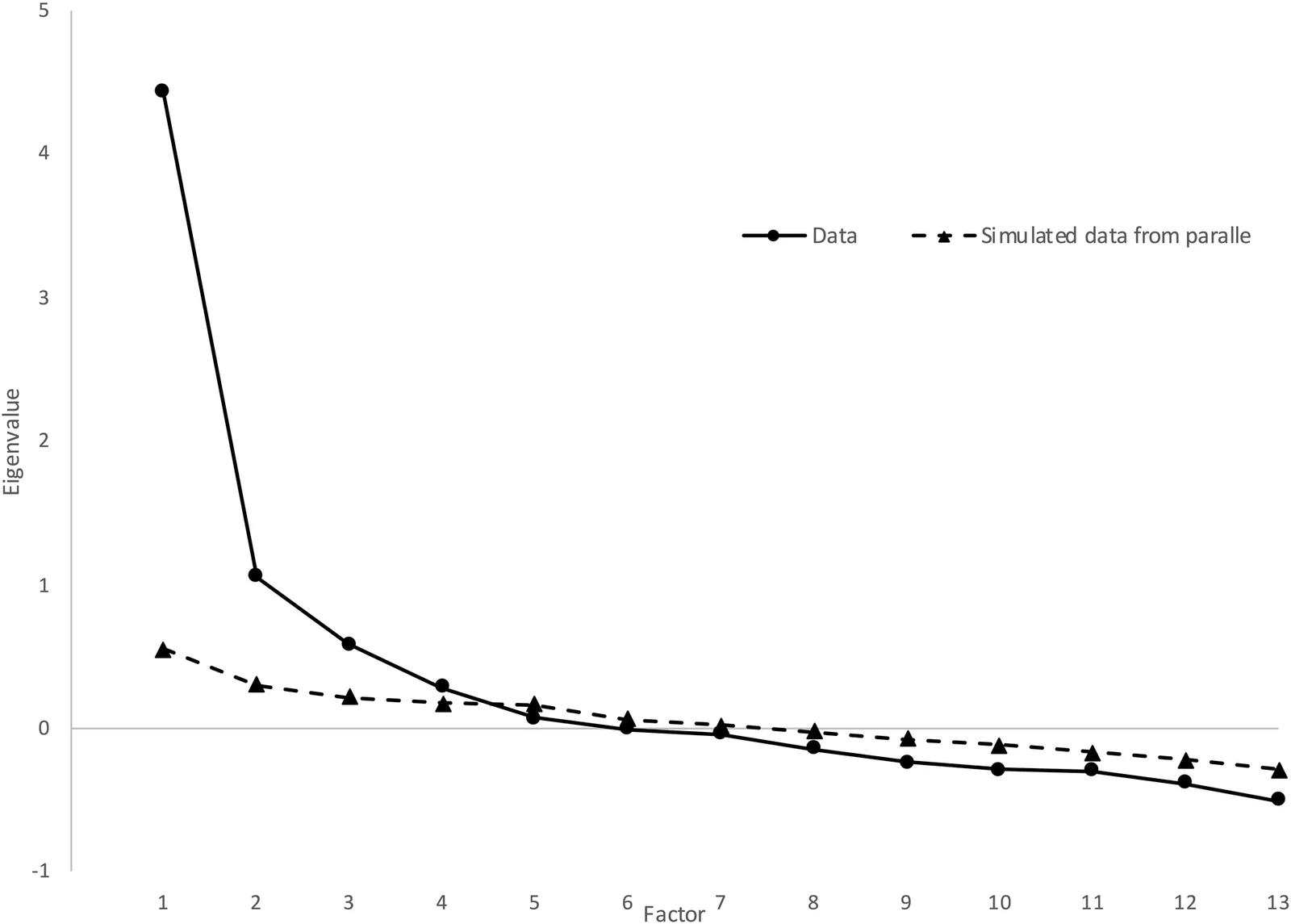
Scree plot with polychoric parallel analysis for factor retention in the EFA sample 1 (*n* = 285).

Floor effects were formally assessed and are reported in Table 3. Three items exhibited high floor effects (> 50%): “avoided smiling or laughing” (63.5%), “avoided talking” (61.0%), and “taken time off work” (58.0%). In contrast, the two items that did not load on any factor, “difficulty pronouncing words” (41.7%) and “missed daycare or preschool” (23.3%), did not show high floor effects, indicating that their failure to load cannot be attributed to restricted range alone.

#### 3.3.3 Confirmatory factor analysis (CFA)

##### 3.3.3.1 Model fit

CFA was conducted to test the four-factor structure identified in the EFA, using WLSMV estimation in R with the lavaan package on Sample 2 (*n* = 286) to account for the ordinal nature of the ECOHIS items. The model included four latent factors: Family Impact (4 items: family member upset, family member felt guilty, family member took time off work, financial impact on the family), Child Self-Image (2 items: avoided smiling/laughing, avoided talking to other children), Child Psychology (2 items: trouble sleeping, irritable/frustrated), and Child Function and Symptoms (3 items: pain, difficulty drinking beverages, difficulty eating certain foods).

The model fit indices reflected a good fit to the data: robust *χ*^2^ = 94.175, df = 38, *χ*^2^/df = 2.48; CFI = 0.979; TLI = 0.969; RMSEA = 0.070 (90% CI: 0.052–0.088); SRMR = 0.049. All fit indices met their pre-specified criteria, as summarized in Table 11.

**Table 11:** Goodness of model fit indices for confirmatory factor analysis on sample 2.

Measure	Value	Target	Interpretation
*χ* ^2^	94.175	—	—
df	38	—	—
χ^2^/df	2.48	<3.0	Good fit
CFI	0.979	>0.90	Good fit
TLI	0.969	>0.90	Good fit
RMSEA	0.070	<0.08	Good fit
RMSEA 90% CI	0.052–0.088	—	—
SRMR	0.049	<0.08	Excellent fit

With *N* = 286, CFA was estimated using WLSMV in R with the lavaan package. Robust χ^2^ and scaled fit indices are reported.

CFI, Comparative Fit Index; df, Degrees of freedom; TLI, Tucker–Lewis Index; RMSEA, Root Mean Square Error of Approximation; χ^2^, Chi-square.

Modification indices were examined to identify potential sources of model misspecification. The largest modification index was observed for the correlated error between PF1 and PF2 (MI = 13.26). A modified model including this correlated error was tested. The modified model demonstrated improved fit: robust *χ*^2^ = 73.619, df = 37, CFI = 0.986, TLI = 0.979, RMSEA = 0.057 (90% CI: 0.038–0.077), and SRMR = 0.045 (Table 12). However, the improvement in fit was modest, and the original model was retained for parsimony.

**Table 12:** Comparison of original and modified CFA models with correlated errors on sample 2 (*n* = 286).

Model	χ^2^	df	χ^2^/df	CFI	TLI	RMSEA	SRMR
Original	94.175	38	2.48	0.979	0.969	0.070	0.049
Modified	73.619	37	1.99	0.986	0.979	0.057	0.045

The modified model included a correlated error between Family member taken time off work and financial impact on the family (MI = 13.26). All fit indices improved in the modified model. CFA = confirmatory factor analysis; MI = modification index.

To evaluate the adequacy of the proposed four-factor structure, we compared it against two competing models: a one-factor model (all 11 items loading on a single OHRQoL factor) and a two-factor model (Child Impact and Family Impact, consistent with the original ECOHIS structure). As shown in Table 13, the one-factor model showed poor fit (CFI = 0.829, TLI = 0.786, RMSEA = 0.184), and the two-factor model also showed poor fit (CFI = 0.881, TLI = 0.848, RMSEA = 0.155). In contrast, the four-factor model demonstrated superior fit (CFI = 0.979, TLI = 0.969, RMSEA = 0.070). All standardized factor loadings were statistically significant (*p* < 0.001) and ranged from 0.681 to 0.967, comfortably above the 0.50 benchmark. The item “avoided smiling or laughing” loaded most strongly onto the Child Self-Image factor (0.967), whereas “frustrated or easily irritated” had the lowest loading (0.681) on the Child Psychology factor. R^2^ values ranged from 0.464 to 0.935, meaning that the proportion of variance explained by the respective latent factors varied from 46.4% to 93.5%. These results are presented in Table 14 and Figure 2.

**Table 13:** Comparison of competing CFA models on sample 2 (*n* = 286).

Model	χ^2^	df	χ^2^/df	CFI	TLI	RMSEA
One-Factor	492.44	44	11.19	0.829	0.786	0.184
Two-Factor	353.40	43	8.22	0.881	0.848	0.155
Four-Factor	94.18	38	2.48	0.979	0.969	0.070

All models were estimated using WLSMV. The four-factor model demonstrated superior fit compared to both competing models.

**Table 14:** Standardized factor loadings, AVE, and CR on sample 2 (*n* = 286).

Factor and Item	Standardized Loading	R^2^	AVE	CR
Factor 1: Family Impact			0.625	0.869
Family member upset	0.789	0.623		
Family member felt guilty	0.831	0.691		
Family member taken time off work	0.805	0.648		
Financial impact on the family	0.733	0.537		
Factor 2: Child Self-Image			0.850	0.919
Avoided smiling/laughing	0.967	0.935		
Avoided talking to other children	0.875	0.766		
Factor 3: Child Psychology			0.551	0.709
Trouble sleeping	0.799	0.638		
Frustrated or easily irritated	0.681	0.464		
Factor 4: Child Function & Symptoms			0.642	0.843
Pain in the teeth, mouth, or jaws	0.798	0.637		
Difficulty drinking beverages	0.846	0.716		
Difficulty eating certain foods	0.758	0.575		

All factor loadings are standardized and significant at *p* < 0.001.

AVE, Average Variance Extracted; CR, Composite Reliability.

**Figure 2:**
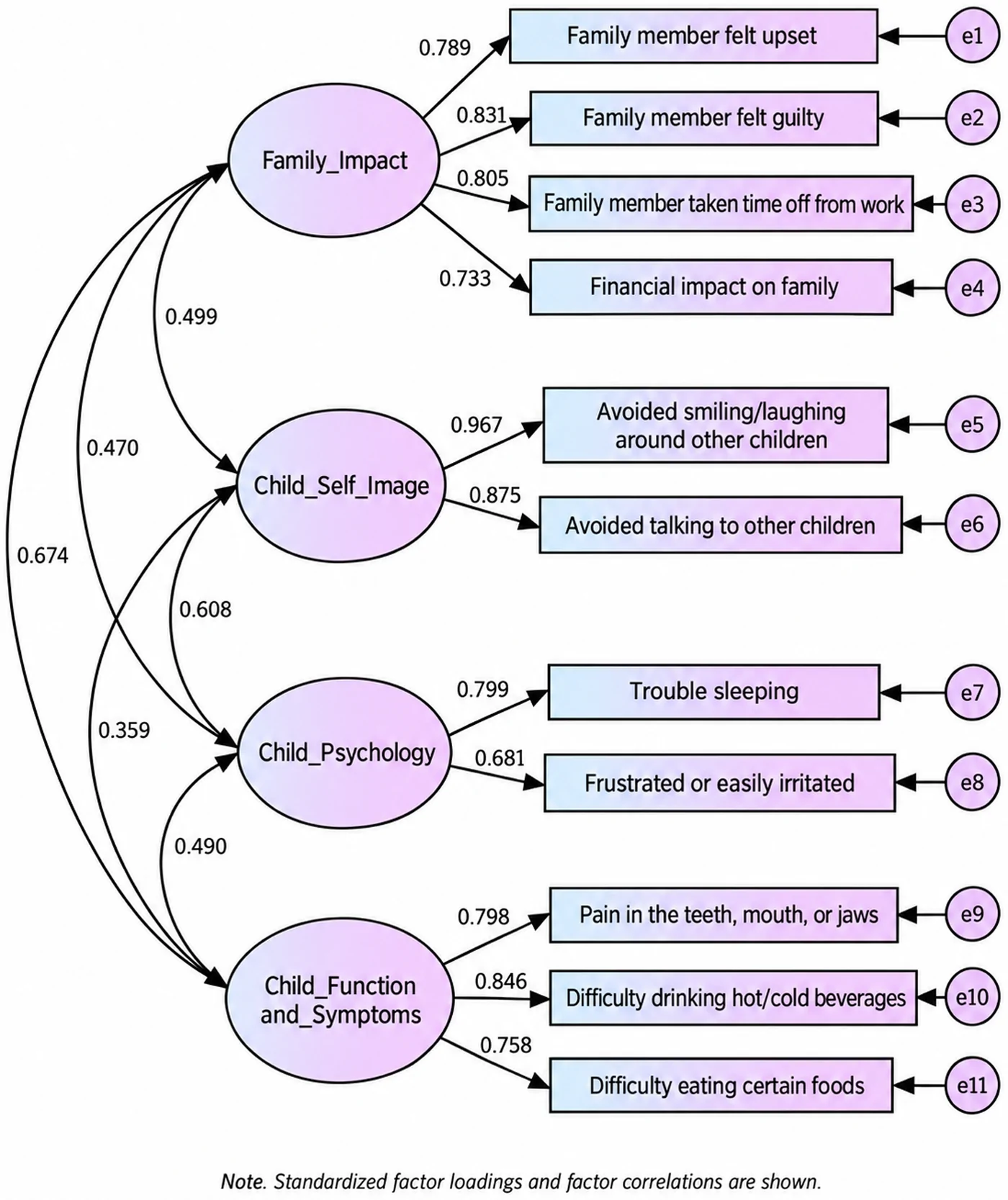
Confirmatory factor analysis of ECOHIS on sample 2 (*n* = 286).

Convergent validity was assessed using the Average Variance Extracted (AVE) and Composite Reliability (CR). AVE values were 0.625 for Family Impact, 0.850 for Child Self-Image, 0.551 for Child Psychology, and 0.642 for Child Function and Symptoms. All AVE values exceeded the recommended threshold of 0.50. CR values were 0.869 for Family Impact, 0.919 for Child Self-Image, 0.709 for Child Psychology, and 0.843 for Child Function and Symptoms (Table 14).

Discriminant validity was assessed using two complementary approaches: the Fornell-Larcker criterion and the HTMT ratio. As shown in Table 15, the square root of the AVE (√AVE) for each factor exceeded the inter-factor correlations, supporting discriminant validity for all factor pairs. Factor correlations ranged from 0.359 to 0.674, indicating that the factors are related but distinct constructs. The strongest correlation was observed between Family Impact and Child Function and Symptoms (r = 0.674), suggesting that children's functional limitations are associated with greater family impact.

**Table 15:** Discriminant validity (fornell-larcker criterion) on sample 2 (*n* = 286).

Factor	AVE	√AVE	Factor 1	Factor 2	Factor 3	Factor 4
Factor 1 (Family Impact)	0.625	**0**.**791**	**1**.**000**	–	–	–
Factor 2 (Child Self-Image)	0.850	**0**.**922**	0.489	**1**.**000**	–	–
Factor 3 (Child Psychology)	0.551	**0**.**742**	0.470	0.608	**1.000**	–
Factor 4 (Child Function and Symptoms)	0.642	**0**.**801**	0.674	0.359	0.490	**1.000**

Discriminant validity is supported when √AVE values exceed the corresponding inter-factor correlations.

Additionally, the HTMT ratios (Table 16) were below the recommended threshold of 0.85 for all the factor pairs (ranging from 0.347 to 0.677). Overall, the CFA supported the four-factor structure of the Arabic ECOHIS, demonstrating good model fit, adequate convergent, and discriminant validity.

**Table 16:** HTMT matrix (heterotrait-monotrait ratio) on sample 2 (*n* = 286).

Factor	Factor 1	Factor 2	Factor 3	Factor 4
Factor 1 (Family Impact)	**1**.**000**	–	–	–
Factor 2 (Child Self-Image)	0.487	**1**.**000**	–	–
Factor 3 (Child Psychology)	0.487	**0**.**611**	**1.000**	–
Factor 4 (Child Function and Symptoms)	**0**.**677**	0.347	0.455	**1.000**

HTMT < 0.85 indicates adequate discriminant validity.

#### 3.3.4 External convergent validity

External convergent validity was assessed using Spearman's rank-order correlation between the total ECOHIS score and the Child Oral Health Rating. A significant positive correlation was observed (Spearman's rho = 0.443, *n* = 553, *p* < 0.001), indicating that higher ECOHIS scores (worse OHRQoL) were associated with poorer perceived oral health. This supports the external convergent validity of the Arabic ECOHIS (Table 17).

**Table 17:** Spearman's correlation between total ECOHIS score and child oral health rating .

Variables	n	Spearman's rho	*p*-value
Total ECOHIS Score vs. Child Oral Health Rating	553	0.443	<0.001

Higher ECOHIS scores indicate worse oral health-related quality of life. Child Oral Health Rating: 1 = Excellent, 2 = Average, 3 = Poor. “I don't know” responses were treated as missing.

## 4 Discussion

This study aimed to assess the factorial validity of the Arabic version of the Early Childhood Oral Health Impact Scale (A-ECOHIS) in a large sample of Saudi mothers of preschool-aged children. We used both exploratory and confirmatory factor analyses, employing a split-sample approach with independent subsamples for EFA and CFA, to examine the underlying structure of the 13-item scale. Our findings revealed a four-factor solution comprising Family Impact (4 items), Child Self-Image (2 items), Child Psychology (2 items), and Child Function and Symptoms (3 items). This structure differs from the original six-domain model proposed by Pahel et al. (12) but aligns with several cross-cultural adaptations, particularly the Farsi version, which also reported a multidimensional structure (19). Importantly, the original two-section (child vs. family) conceptualization was not fully supported, as seven of the nine child items loaded onto the child-related factors, and two items (pronunciation difficulty and missed school) did not load on any factor.

### 4.1 Factor structure and comparison with other language versions

The four-factor solution accounted for 57.76% of the total variance, which is higher compared to other ECOHIS validation studies (e.g., 48.1% in the Brazilian version (15) and 45.6% in the Malay version (18). The Family Impact factor demonstrated strong loadings (0.733–0.831) and acceptable internal consistency (*α* = 0.794, *ω* = 0.796). However, its AVE was 0.625, which met the recommended threshold of 0.50. This suggests that the four family impact items share a common construct. Similar findings have been reported in other language validations where family impact items sometimes cross-load or show heterogeneous content (15, 18).

The Child Self-Image factor (two items: avoided smiling/laughing and avoided talking) showed good internal consistency (*α* = 0.831, *ω* = 0.832) and good convergent validity (AVE = 0.850, CR = 0.919). In the polychoric EFA, the avoided-talking item produced an ultra-Heywood estimate, suggesting local instability in the exploratory solution; however, its standardized loading in the independent CFA sample was within the admissible range (0.875). The high factor loading for “avoided smiling or laughing” (0.967) with an R^2^ of 0.935 indicates strong alignment with the latent construct. While this demonstrates strong measurement properties, it also raises the possibility of local dependency or redundancy. However, removing the item would leave the self-image factor with only a single indicator, which is psychometrically undesirable. Therefore, retaining both items is recommended for content coverage.

The Child Psychology factor (two items: trouble sleeping and irritable/frustrated) was a distinct factor in our solution, with loadings of 0.799 and 0.681, respectively. This factor had questionable internal consistency (*α* = 0.614, *ω* = 0.611) but acceptable convergent validity (AVE = 0.551, CR = 0.709). The relatively lower reliability for this factor suggests that these two items may not fully capture the breadth of the psychological impact construct, and future research may benefit from adding items to this domain.

The Child Function and Symptoms factor combined three items: difficulty drinking beverages (loading 0.846), difficulty eating certain foods (0.758), and pain (0.798). This factor had an acceptable AVE (0.642) and composite reliability (CR = 0.843). Notably, this factor merges the original symptom domain (pain) with two function items (drinking and eating), suggesting that for Arabic-speaking mothers, the experience of pain is closely linked to functional limitations in eating and drinking. This is clinically plausible because pain often manifests during meals or when consuming hot/cold beverages.

### 4.2 Items that did not load

Two items did not load on any factor (loadings < 0.40): difficulty pronouncing words and missed daycare or preschool. The failure of these items to load is consistent with several other ECOHIS validation studies, in which certain function items often exhibit low loadings or cross-loadings (15, 18). Pronunciation difficulty may be less salient because many preschool children are still developing speech, and parents may not associate articulation problems with oral health unless anterior teeth are visibly missing. This item may be more relevant in older children or those with severe caries affecting front teeth.

Missed school due to dental problems was the most frequently reported impact (43.5% reported sometimes or more). The item did not load substantially on any factor despite its relatively frequent selection. This suggests that school absence may have more context-dependent consequences of oral health problems that do not correlate strongly with other child function items in this sample

A formal assessment of floor effects (Table 3) revealed that three items exhibited high floor effects (> 50%): “avoided smiling or laughing” (63.5%), “avoided talking” (61.0%), and “taken time off work” (58.0%). Despite this limited variability, these items still demonstrate moderate to strong factor loading in EFA, suggesting that the floor effects did not substantially weaken their factorial performance. In contrast, the two items that did not load on any factor, “difficulty pronouncing words” (41.7%) and “missed daycare or preschool” (23.3%), did not show high floor effects, indicating that their failure to load cannot be attributed to restricted range alone. Cultural and contextual factors may better explain their non-loading.

### 4.3 Confirmatory factor analysis and model fit

The CFA on the independent Sample 2 (*n* = 286) supported the four-factor structure with good fit indices using WLSMV estimation: robust *χ*^2^ = 94.175, df = 38, *χ*^2^/df = 2.48; CFI = 0.979; TLI = 0.969; RMSEA = 0.070 (90% CI: 0.052–0.088); SRMR = 0.049. All fit indices met their respective pre-specified criteria. With *N* = 286, the *χ*^2^ statistic is often significant; therefore, CFI and TLI are more reliable indicators of model fit.

Modification indices were examined, and a correlated error between PF1 and PF2 (MI = 13.26) was tested. The modified model demonstrated improved fit: robust *χ*^2^ = 73.619, df = 37, CFI = 0.986, TLI = 0.979, RMSEA = 0.057 (90% CI: 0.038–0.077), and SRMR = 0.045. However, the improvement in fit was modest, and the original model was retained for parsimony.

Competing models were tested to evaluate the adequacy of the proposed four-factor structure. The one-factor model showed poor fit (CFI = 0.829, TLI = 0.786, RMSEA = 0.184), and the two-factor model consistent with the original ECOHIS structure also showed poor fit (CFI = 0.881, TLI = 0.848, RMSEA = 0.155). In contrast, the four-factor model demonstrated superior fit, (CFI = 0.979, TLI = 0.969, RMSEA = 0.070). External convergent validity was further supported by a significant positive correlation between the total ECOHIS score and Child Oral Health Rating, which indicates that higher ECOHIS scores (worse OHRQoL) are associated with poorer perceived oral health, consistent with the original validation (12) and other language versions (13–19).

Discriminant validity was supported using both the Fornell-Larcker criterion (√AVE values exceeded inter-factor correlations for all factor pairs) and the HTMT ratio (all values < 0.85 for all the factor pairs). Factor correlations ranged from 0.359 to 0.674, indicating that the factors are related but distinct constructs. These findings indicated adequate distinction among all the four factors in this sample. The strongest correlation was observed between Family Impact and Child Function and Symptoms (r = 0.674), suggesting that children's functional limitations are associated with greater family impact.

### 4.4 Reliability

The total scale demonstrated good internal consistency (*α* = 0.825, *ω* = 0.827), and the Family Impact section (*α* = 0.794, *ω* = 0.796) and Child Self-Image domain (*α* = 0.831, *ω* = 0.832) had acceptable to good reliability. The Child Psychology domain had questionable reliability (*α* = 0.614, *ω* = 0.611), and the Child Function domain had poor reliability (*α* = 0.590, *ω* = 0.645). This is not surprising given that the Child Function domain includes heterogeneous items (drinking, eating, pronunciation, school absence) that may not form a unidimensional scale. In the EFA, only two of the four original function items (drinking and eating) loaded with pain on a single factor; pronunciation and missed school did not load. Therefore, these findings suggest caution when interpreting the original Child Function subscale separately.

### 4.5 Strengths and limitations

This study has several strengths. It is the first to perform both EFA and CFA on the Arabic ECOHIS using a split-sample approach, with EFA conducted on one independent subsample (*n* = 285) and CFA on a separate subsample (*n* = 286), providing a rigorous test of factorial validity. The total sample (*N* = 602) exceeded the minimum required sample size for factor analysis. The use of a polychoric correlation matrix with PAF and Oblimin rotation allowed the ordinal nature of the ECOHIS responses to be considered throughout the exploratory analysis. The CFA included multiple fit indices, AVE, CR, and discriminant validity assessments (Fornell-Larcker and HTMT). The sample was diverse in terms of children's ages, genders, maternal education levels, and geographic regions within Saudi Arabia.

Several limitations must be acknowledged. First, although we used a split-sample approach for EFA and CFA, this internal cross-validation does not replace external validation. The four-factor structure identified in the present study, based on 11 retained items from the original 13-item A-ECOHIS, should therefore be regarded as provisional and it requires further investigation it can be considered a validated alternative to the original 13-item A-ECOHIS. Future studies should recruit independent samples from different settings and populations to externally validate the proposed four-factor, 11-item structure and to determine whether the observed psychometric properties are reproducible. Second, the study relied on convenience and snowball sampling via social media, which may have introduced selection bias (e.g., over-representation of mothers with higher education, as 64.8% held a bachelor's degree), future studies should use stratified random sampling to capture a more diverse demographic profile. Third, the low Cronbach's alpha for the Child Function domain (0.590) and the questionable reliability for the Child Psychology domain (0.614) suggest that these subscales may benefit from item revision in future studies. Fourth, the child age range (2–6 years) included some children below the typical preschool age, but this reflects real-world heterogeneity. Fifth, the study did not include test-retest reliability, although this was previously established by Farsi et al. (20). Sixth, clinical caries data were available only for a subset of participant (*n* = 150) and were not incorporated into this psychometric analysis, therefor, criterion-related validity against objective caries measures (e.g., dmft scores) was not evaluated. Finally, the two items that did not load (pronunciation difficulty and missed school) may require cultural adaptation or re-wording for Arabic-speaking populations.

## 5 Conclusion

The Arabic version of the Early Childhood Oral Health Impact Scale (A-ECOHIS) demonstrated a four-factor structure comprising Family Impact, Child Self-Image, Child Psychology, and Child Function and Symptoms in a large sample of Saudi mothers, using a rigorous split-sample approach with independent subsamples for exploratory and confirmatory factor analyses. The total scale and the Child Self-Image subscale demonstrated good reliability, whereas the Family Impact subscale showed acceptable reliability. The Child Psychology subscale demonstrated questionable reliability, highlighting the need for further psychometric refinement of this domain.

The four-factor model demonstrated acceptable fit in confirmatory factor analysis, with adequate convergent and discriminant validity. The original 13-item A-ECOHIS remains a useful instrument for measuring OHRQoL in Arabic-speaking preschool children, with the caveat that users should be aware of its multidimensional structure and the need for further psychometric refinement, particularly for the Child Psychology factor and the two items not retained. Resulting in a provisional 11-item, four-factor model. This reduced structure should be considered preliminary rather than a newly validated version of the A-ECOHIS, as its psychometric properties and factor structure have not yet been confirmed in an independent sample. External validation using a newly recruited and independent sample is therefore warranted before the 11-item model can be considered a validated alternative to the original 13-item A-ECOHIS.

## Data Availability

The raw data supporting the conclusions of this article will be made available by the authors, without undue reservation.
